# Mr. Brodie on Aneurisms by Anastomosis

**Published:** 1829-10-01

**Authors:** 


ANEURISM BY ANASTOMOSIS CURED BY THE APPLICATION OF LIGATURES. By B. C. BRODIE, F.R.S. Surgeon to the King, &c.

In the fifteenth volume of the Medico-Chirurgical Transactions, very recently published, is a short account of a case of the above description by Mr. Brodie. As it illustrates what we believe to be an important point of surgery, we hasten to give an authentic statement of the facts, although we have alluded to the case before in an article on pulsating tumours of the scalp.

Miss ——, in the year 1809, being then five years of age, struck her forehead very severely against the corner of a bed-post. Shortly afterwards a small pulsating tumour, the size of a pea, was observed at the stricken part, which produced little inconvenience, and acquired but little augmentation of size till 1821, when the young lady consulted a surgeon in London, who attempted the cure by pressure with compresses and bandage. Constant and severe pain were the consequences of the treatment, which, so far from proving of service, appeared to accelerate the growth and increase the pulsation of the tumour. From this time, also, there were frequent attacks of intense headache, which could only be relieved by blood-letting. The local treatment was now intermitted till 1824, when the size of the tumour again augmenting, another attempt at pressure was made by Sir Astley Cooper, with no better success than the former. In the latter part of June, 1826, the disease having made still further progress, Sir Astley was again consulted, and now applied a ligature, at four different times, round each of four principal arteries by which the tumour was supplied. A slight diminution in its size ensued; but the favourable change was of short duration, for, in the Winter of 1827, both the bulk and the painful sensations were greater, whilst a constant sense of weight over the eyes was experienced, and much dejection of spirits. Occasionally there were paroxysms of pain still more violent than usual, which were followed by a state of extreme languor and exhaustion.

"Miss —— remained precisely in this state, except that the tumor continued slowly to enlarge, until the 9th of October, 1828, when she arrived in London, after an absence of many months, and I saw her, in consultation with Dr. Robertson, of Northampton. The tumor was now bigger than a large double walnut, occupying a spot on the right side of the forehead, immediately below the margin of the hairy scalp. When the fingers were applied to it, they received an impression as if it was composed of a mass of tortuous vessels, and a strong pulsation was perceptible in every part of it. The skin covering the tumor was thin, and on some occasions, as in coughing, when the vessels were unusually distended, it appeared as if on the point of bursting. When the scalp was shaved, large and tortuous arteries were to be seen, even from a considerable distance, passing into the basis of the tumor, in every direction, from each temple, from the orbit of the right eye, and over the crown of the head from the occiput. Pressure being made on the two temporal arteries at the same instant, the pulsation of the tumour was perceptibly, but not greatly, diminished. There was a constant sense of weight and pain in the forehead, and the latter was very much aggravated by pressure on the tumor, especially on a particular spot towards its upper edge.

"The sufferings of the patient were such that she was willing to submit to any plan of treatment which might afford her even a chance of being relieved. On considering the subject, it appeared to Dr. Robertson and myself that there was no reason to expect advantage from any further attempt to obliterate the arteries by which the tumor was supplied with blood, nor indeed from any operation which had not for its object the complete extirpation and removal of the diseased structure. But the attempt to accomplish this object by means of the knife, would necessarily be made at the risk of a most alarming hæmorrhage, and the application of the actual cautery or of caustic would not only be uncertain as to the result, but, if carried to a sufficient extent completely to answer the intended purpose, might occasion such injury to the bone and periosteum, as would be productive of much subsequent inconvenience, if not actual danger, to the patient. Under these circumstances, Dr. Robertson immediately assented to the proposal which I made, that I should endeavour to extirpate the tumour by means of ligatures, so applied as to produce the complete strangulation of it at its base. There seemed at any rate to be no more effectual, nor any safer method of proceeding, but even with respect to this, it was impossible not to experience in the first instance considerable apprehensions as to the loss of blood, which might take place on the separation of the slough. These apprehensions were however greatly diminished, if not altogether removed, in consequence of the conviction which we felt, that the unusual dilatation of the principal arteries of the scalp, was to be regarded as the effect, and not the cause, of the morbid growth of the smaller vessels, and as being likely to subside immediately on the tumor being destroyed."

After a further consultation with Mr. Keate, Sir Astley Cooper and Dr. Robertson, Mr. Brodie performed the operation on the 15th of October.

"A long steel needle, the length of which was about double the diameter of the tumor, was passed between it and the periosteum, penetrating the skin on each side. By means of this needle the tumor was raised as much as possible, and a second needle was introduced in the same manner, but beneath, and at right angles to, the first. A very strong silk ligature was then bound several times round the base of the tumor below the needles, as tight as it could be drawn. The tumour immediately assumed a purple color, as if in a state of strangulation. The operation occasioned great pain both at the time and afterwards; but from the instant of the ligature having been applied, the peculiar sufferings occasioned by the disease were at an end."

In the evening the patient required bleeding, but, on the 18th, all the arteries entering the tumour had either ceased to pulsate, or did so feebly, with the exception of those at the upper part. Concluding that the strangulation was not every where complete, Mr. Brodie armed one of the needles with a strong double ligature, then drew it through, and, having removed the needle, tied the ligatures one on each side. On the 20th, the same thing was done with the other needle, and, on the 26th, the included tumour sloughed away altogether, without a drop of hæmorrhage. The ulcer soon put on a healthy aspect, and the nitric acid removed some spots upon its surface, that appeared to evince a disposition to re-produce the original mass. By the 2nd of December the cicatrix was completely formed, and no remains of the tumour existed. The arteries which previously were so much enlarged, now pulsated with no more force than those on the opposite side, and the patient was free from all inconvenience.

We consider the preceding case as particularly interesting in a surgical point of view, and it goes to prove the correctness of a principle on which we have often and strenuously dwelt. We allude to the error of viewing these cases of pulsating tumour as analogous to aneurism, and, therefore to be treated like that disease. Suppose that in this, as in two other cases that have happened within these few years, the carotid artery had been tied! Would it not have been a cruel and unjustifiable operation, an operation which, if it failed to kill, would almost certainly fail to cure? We think there can be but one answer to the question, and, more than that, we venture to predict that a better æra has dawned upon this part of surgical science, and that none, in future, will dream of resorting to any such attempts. We are not without our share of satisfaction, at finding that the views which we have all along maintained are proving to be correct, and that our published sentiments respecting the nature and treatment of this disease do not differ from those which are beginning to be entertained by the best informed members of the profession.

